# The nationwide Finnish anticoagulation in atrial fibrillation (FinACAF): study rationale, design, and patient characteristics

**DOI:** 10.1007/s10654-021-00812-x

**Published:** 2022-01-05

**Authors:** Mika Lehto, Olli Halminen, Pirjo Mustonen, Jukka Putaala, Miika Linna, Janne Kinnunen, Elis Kouki, Jussi Niiranen, Juha Hartikainen, Jari Haukka, Kari Eino Juhani Airaksinen

**Affiliations:** 1 Department of Internal Medicine, Lohja Hospital, Helsinki and Uusimaa Hospital District, Lohja, Finland; 2 Heart and Lung Center, Helsinki University Hospital and University of Helsinki, Helsinki, Finland; 3 Aalto University, Espoo, Finland; 4 Turku University Hospital and University of Turku, Turku, Finland; 5 Department of Neurology, Helsinki University Hospital and University of Helsinki, Helsinki, Finland; 6 University of Eastern Finland, Kuopio, Finland; 7 Florida State University, Tallahassee, FL USA; 8 Kuopio University Hospital and University of Eastern Finland, Kuopio, Finland; 9 University of Helsinki, Helsinki, Finland

**Keywords:** Atrial fibrillation, Register study, Anticoagulation, Cost-effectiveness, Stroke

## Abstract

Atrial fibrillation (AF) is a major cause of ischemic stroke and the number of AF patients is increasing. Thus, up-to-date multifaceted data about the characteristics of AF patients, their treatments, and outcomes are urgently needed. The Finnish anticoagulation in atrial fibrillation (FinACAF) study has collected comprehensive data on all Finnish AF patients from 1st January 2004 to 31st December 2018. The aim of this paper is to describe the study rationale, the process of integrating data from the applied resources and to define the study cohort. Using national unique personal identification number, individual patient data is linked from nationwide health care registries (primary, secondary, and tertiary care), drug purchases, education, and socio-economic status as well as places of domicile, incomes, and taxes. Six regional laboratory databases (~ 282,000, 77% of the patients) are also included. The study cohort comprises of a total of 411,000 patients. Since the introduction of the national primary care register in 2012, 9% of all AF patients were identified outside hospital care registers. The prevalence of AF in Finland—4.1% of whole population—is for the first time now established. The FinACAF study allows a unique possibility to investigate the epidemiology and socio-medico-economic impact of AF as well as the cost effectiveness of different AF management strategies in a completely unselected, nationwide population. This article provides the rationale and design of the study together with a summary of the characteristics of the cohort.

**Supplementary Information:**

The online version contains supplementary material available at 10.1007/s10654-021-00812-x.

## Background

Atrial fibrillation (AF) is the most common sustained arrhythmia. It is associated with a wide spectrum of symptoms, impairment of quality of life and complications, and the prevalence of AF in increasing. It has been estimated that the prevalence of AF in adult population in Europe is about 2.1% [1] and incidence of AF is about 1.3 per 1000 person-years [2]. These figures are predicted to increase about twofold until year 2050. While for age of 55 years, the lifetime risk of AF is more than one out of three, it also is the major cause of stroke, and a vast majority of AF patients have an indication of oral anticoagulation (OAC) [3, 4].

The population in Finland is approximately 5.5 million, and the country is divided into five university hospital districts. The health care system is public Beveridge-type model. Approximately 15–20% of health care is funded directly by households and 70–75% by taxation. The health care system is grounded on the primary health care center network instituted by municipals or consortiums of municipals.

### Health registers in Finland

The national health care registries in Finland include two separate registers upheld by registry of Finnish Institute for Health and Welfare (THL): (1) Finnish Care Register (HILMO) which includes information of all secondary and tertiary inpatient (hospitalizations and procedures) and outpatient care, including also scheduled and emergency care specialist visits, and (2) Finnish Care Register (AvoHILMO) that includes all primary health care contacts (e.g. visits to general practitioners and nurses) at health centers. Finland and Taiwan are probably the only countries where the nationwide health care register includes comprehensive information also from primary health care [5]. HILMO register contains nationwide data of all inpatient hospital discharges with personal identification number since 1969. AvoHILMO register was introduced in 2011 and was completed during 2012. Notably, a minority of AF patients are treated solely by private practitioners. Most of such AF patients can be identified based on the reimbursed medications for AF from National Reimbursement Register upheld by Social Insurance Institute (KELA).

The register of social care (SosiaaliHILMO), also maintained by THL, collects information on institutional and housing services of social care provided for the elderly and the disabled. This nationwide register includes information on service provider, type and quantity, and the provision of the service and the need for service of the social care clients. SosiaaliHILMO register was established in 1995, and the data content and classifications have not markedly changed since then.

### Need for a comprehensive register study of atrial fibrillation

OACs are highly effective in decreasing the risk of stroke in AF patients, but vitamin K antagonists (VKA) need lots of costly health care resources. It has been estimated that annual costs for service provider are approximately 940 € higher in warfarin users compared to non-users in Finland [6]. Hence, it is crucial to have up-to-date, comprehensive data on the outcomes of OAC therapy in AF patients during the era of direct oral anticoagulants (DOACs). This information is urgently needed for “leading with data” in the changing world of medico-economical arrangements.

### Objectives of the study

It has been estimated that the number of AF patients in Finland is around 150,000. However, aggregation of data from all available public registers have not been previously performed in Finland. Therefore, we aimed to construct a nationwide registry of all patients with AF—also those treated solely in primary care—compiling all identifiable individuals in the separate registers between 2004 and 2018. In this cohort, we aim to analyze all aspects of current AF treatment practices with our main focus being on OAC treatment and its consequences. Data on quality of warfarin treatment is measured as time in therapeutic range (TTR) through INR monitoring, and the patient adherence and persistence during DOAC treatment is evaluated from the National Reimbursement Register (KELA).

At present, there is limited data on the impact of socio-economic factors on AF treatment. The present study includes information on taxable incomes and education of the patients [7]. Because all the available contacts with the health and social care institutions and organizations are evaluated, this database allows a unique possibility to investigate all costs and cost effectiveness in relation to different AF treatments.

The primary objectives are to investigate the risk of stroke, systemic thromboembolism, bleeding events and myocardial infarction as well as all-cause and cardiovascular death among patients with AF and various subgroups in relation to different OAC treatments—including DOAC and warfarin treatment with the data of different TTR levels—and in patients with no OAC treatment.

The secondary and exploratory objectives include e.g. investigating the use and costs of health care services and cost effectiveness in relation to different treatments, relations of socio-economic status, mental illness and dementia with the treatments the patients are given, and the associations of these factors with the major outcomes. The laboratory and ECG data will be applied as adjusting variables, but for example, changes in hemoglobin and renal function will also be studied as endpoints. The complete list of the objectives of the study protocol is provided in the “Online Appendix”.

## Methods

**Fig. 1:**
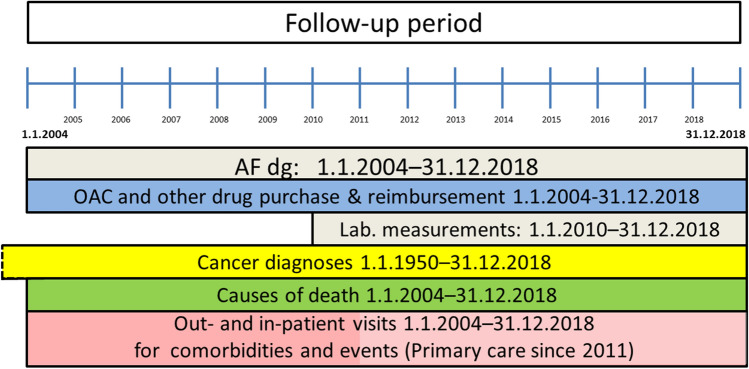
Schematic presentation of data collection periods. AF, atrial fibrillation; OAC, oral anticoagulation

The FinACAF study cohort comprises of all AF patients gathered from national health care registers (hospitalizations and outpatient specialist visits: HILMO, and primary health care: AvoHILMO and National Reimbursement Register upheld by Social Insurance Institute (KELA). Inclusion criteria was an ICD-10 diagnosis code I48 for AF between 01st January 2004 to 31st December 2018 in any of the above-mentioned registers. All Finnish inhabitants have a unique national identification number, and the patients’ individual data from Finnish nationwide population registers and regional laboratory databases were linked together, using this identification code. Pseudonymization of patient identification numbers was performed by KELA, and the research group received individualized, but unidentifiable data. All patient data handled by the researchers are therefore pseudonymized, which ensures full data protection of the patients. Figure 1. depicts the data collection periods based on the applied registers and Table 1. lists all the national registers linked to the AF cohort.

**Table 1:** Registers used in the study

Register	Registry	Information obtained
Finnish Care Register for Health Care (Hospital/HILMO)	Finnish Institute for Health and Welfare	Diagnoses (ICD-10) and interventions (NCSP) with codes
Finnish Care Register, (Primary/AvoHILMO)	Finnish Institute for Health and Welfare	Diagnoses (ICD-10, ICPC-2) and interventions (NCSP) with codes
National Prescription Register	The Social Insurance Institution of Finland (KELA)	Drug purchases (date, ATC codes, amount)
National Reimbursement Register	The Social Insurance Institution of Finland (KELA)	Reimbursements for drug purchases and for private health care expenses
National Causes of Death Register	Statistics Finland	Death dates and causes of deaths
National Cancer Registry	Finnish Cancer Registry	ICD-O-3 codes, date of diagnosis and other information
Laboratory databases	Six largest central laboratories in Finland	INR and other relevant laboratory measurements
Population Register	Population Register Center	Places of domicile
Finnish Care Register for Health Care (Social care/SosiaaliHILMO)	Finnish Institute for Health and Welfare	Non-hospital institutionalizations
Tax Register	Tax Administration	Income and taxes
The Register of Completed Education and Degrees	Statistics Finland	Education and socio-economic status

ICD-10: International Classification of Diseases 10th Revision; NCSP: Nordic Classification of Surgical Procedures [23]; ICPC-2: International Classification of Primary Care2nd Revision. HILMO: hospitalizations and outpatient specialist visits; AvoHILMO: primary health care; and KELA: National Reimbursement Register upheld by Social Insurance Institute

The diagnoses of comorbidities (“Online Appendix”) were constructed in a hierarchal manner using data in the following sequence: (1) ICD-10 diagnoses from HILMO or AvoHILMO; (2) ICPC-2 entries of primary care visits (AvoHILMO); (3) entitlement to reimbursement for the comorbidity or disease (KELA); and (4) prescribed medication for the comorbidity (KELA). The details of obtaining comorbidity diagnoses are presented in the “Online Appendix”.

**Fig. 2:**
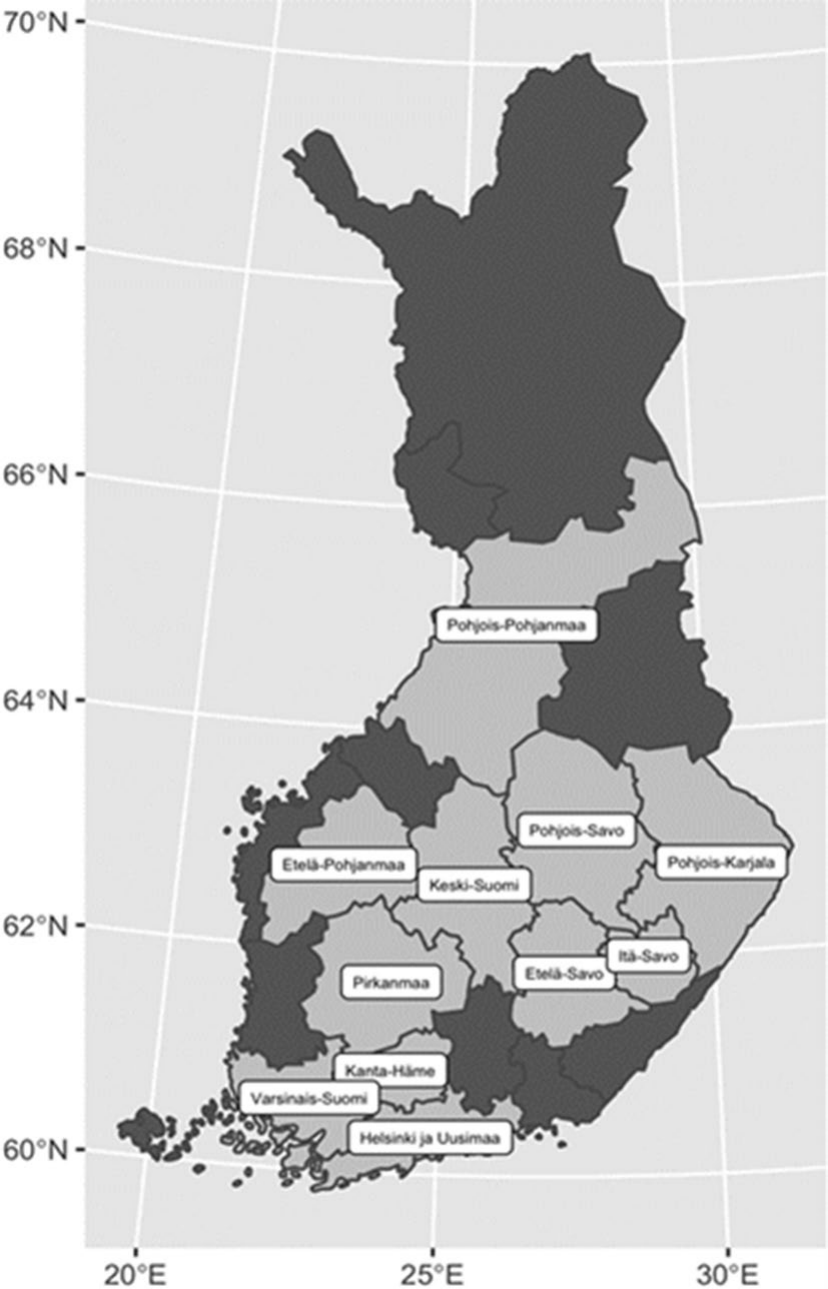
The included hospital districts with laboratory data

Laboratory data from 01st January 2010 to 31st December 2018 were available from the six largest central laboratories with a total catchment population of 4.2 million (77% of the Finnish population; Fig. 2). There were laboratory data entries from a total of 282,000 patients.

### The permissions and the registrations of the study

The study has been registered into the ENCePP e-register (http://www.encepp.eu; EUPAS29845) and in to the Clinicaltrials.gov/ct2/home (NCT04645537). The study has been approved by the Ethics Committee of the Medical Faculty of Helsinki University, Helsinki Finland (nr. 15/2017) and granted research permission from the Helsinki University Hospital (HUS/46/2018). Respective permissions were obtained from the KELA (138/522/2018), THL (THL/2101/5.05.00/2018), Population Register Centre (VRK/1291/2019-3), Statistics Finland (TK-53-1713-18/u1281) and Tax Register (VH/874/07.01.03/2019).

Our study is entirely based on registry data without patient contacts in any phase of the study. Thus, no patient consents are needed according to Finnish legislation. All patient data were pseudonymized, ensuring full data protection of the patients according to European General Data Protection Regulation (EGDPR).

### Description of the cohort

**Fig. 3:**
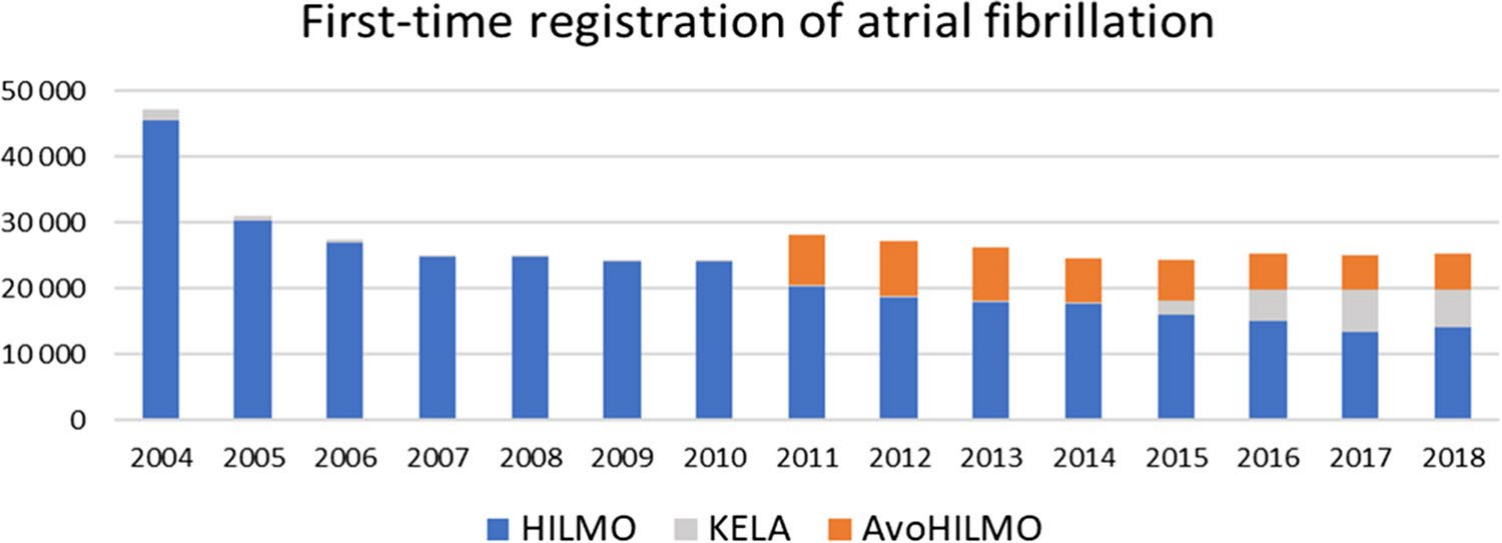
First-time registrations atrial fibrillation according to different national registries. The graph depicts the yearly distribution of first-time registrations of atrial fibrillation. HILMO, hospitalizations and outpatient specialist visits; AvoHILMO, primary health care; and KELA, National Reimbursement Register upheld by Social Insurance Institute

Between years 2004 and 2018, a total of 411 080 patients were identified to have a diagnosis of AF in Finland. As expected, the highest number of new diagnoses were found during 2004 when most of the patients with previously diagnosed AF were also listed for the first time in the study register. Similarly, during 2011–2013 there was an abrupt increase of new AF diagnoses when the primary care data from AvoHILMO was for the first time captured. Figure 3 illustrates the compilation of the cohort from separate registers. The inclusion of primary care registers (AvoHILMO) and drug prescription/reimbursement data (KELA) increased the number of patients by 15,783 (9%) during 2012–2018 compared to the hospital discharge registry (HILMO).

The total number of AF patients on 31st December 2018 was 227,114, which translates to an AF prevalence of 4.1% in Finland (population of 5,517,900).

Comorbidities in the entire cohort are presented in the “Online Appendix”. The patients with very early entry to the cohort had shor—if any—history collection period before the entry date and this caused underestimation of comorbidities during the early cohort entry. Table 2 provides baseline characteristics of the 178,253 new AF patients at the entry to the study cohort between years 2012 and 2018.

**Table 2:** Baseline characteristics of the new atrial fibrillation patients at the time of entry to the study cohort between years 2012 and 2018

	n (%, of the total 178,253 patients)
Female	87,165 (49%)
Age, mean (± SD), median; years	73.7 (± 12.6), 75
Age ≥ 65 years	140,093 (79%)
Age ≥ 75 years	89,860 (50%)
Hypertension	146,115 (82%)
Diabetes	44,171 (25%)
Stroke or TIA	30,312 (17%)
Heart failure	32,627 (18%)
Vascular disease^a^	51,080 (29%)
Hyperlipidemia	96,834 (54%)
CHA_2_DS_2_-VASc, mean (± SD), median	3.8 (± 1.8), 4

TIA, transient ischemic attack

^a^Coronary artery disease or peripheral artery disease. CHA_2_DS_2_-VASc: congestive heart failure, hypertension, age ≥ 75 years, diabetes mellitus, stroke, vascular disease, age 65–74 years, sex category (female)

## Discussion

The purpose of this nationwide register study is to collect all the available register data on AF patients in Finland. The primary aims are to study risk of stroke, systemic thromboembolism, bleedings and myocardial infarction as well as mortality among AF patients in different patient groups in relation to OAC treatment. Taking also in consideration DOAC and warfarin treatment with the data of different TTR levels—as well as patients without OAC treatment.

Furthermore, in the substudies we aim to assess the cost-effectiveness of different treatments and diagnostic studies together with socio-economical profile and clinical endpoints.

The national specialist care register HILMO is well-validated, and the number of studies assessing the completeness and accuracy of the HILMO data is remarkable [8]. For example, the starting point of this kind of register—accurate input of national identification numbers in the Finnish Hospital Discharge Register has been as high as 99.5% during the early 2000s [8]. Secondly, especially in the cardiovascular diseases the diagnostic accuracy has been very high [8, 9].

The more novel register, the primary care register AvoHILMO is only recently documented and validated [10, 11]. When analyzing the prevalence of health risks, like elevated blood pressure or overweight, primary care register does not work properly, and the data transfer from the local information systems to the national AvoHILMO register could be better [11]. Validity of the diagnosis of AF has not been thus far evaluated from the AvoHILMO register. However, in daily practice the diagnosis of AF in primary care is always based on an ECG, and automated ECG analyses to confirm the finding are widely utilized.

During the planning phase based on our previous experience, we observed the incompleteness of any of the used registers alone and need to combine information from all the available registers to have complete reliable data on Finnish AF patients [12]. For instance, diagnoses of hypertension are incompletely recorded in specialist care (HILMO), but more often documented in primary care register (AvoHILMO), and the evidence of hypertension was completed with the medication data from the National Reimbursement Register upheld by registry KELA. On the other hand, stroke was almost always recorded in specialist care. These observations emphasize utilization of all the available data from all available sources, in particular when a multifaceted disease like AF is studied.

All the Nordic countries have to some extent similar health care structures which are based on tax-funded public health care. Furthermore, in all Nordic countries, citizens have an individual identification number, which makes it possible to combine national register data. Nationwide register studies of AF have been published from Nordic countries merging also data from several separate registers [13–15]. However, these countries do not have a national primary health care registry, and nationwide studies have been based only on cases found in the hospital setting. From Sweden, it has estimated that 22% of AF patients are treated only in primary care practices [13]. In our study 9% of patients would not been found to have AF if only hospital-based registers would have been available.

Primary health care register was introduced in 2011, and during the era of the DOACs, the registration of patients as new AF patients on the basis of the drug reimbursement register has changed the establishment of registration of the initial diagnosis in the 2010s. Thus, the role of specialized health care has been declining.

### Information content of the study

The unique feature of FinACAF study is that it combines information on all contacts of AF patients with the public Finnish health care and social care systems. This data allows answering for unlimited number of questions and thorough evaluation of the needs of care, risk of various endpoints as well as total costs of the treatments, endpoints, and complications. The long (15 years) study period enables several evaluations of trends and changes regarding treatments and their gains.

The extent of laboratory information with the timespan of 9 years in FinACAF is vast. The planned analyses include e.g. assessment of renal function as well as hemoglobin and platelet count, and with this information the changes in the laboratory values during the follow-up can be analyzed. TTR of the patients with warfarin opens the window to analyze balance of the treatment with warfarin, and this information will be used to compare these patients with the patients on DOACs or without any OAC. During the era of DOACs, the reports so far published including the data on TTR have had very limited number of patients [16, 17]. When including patients who were withhold of any OAC therapy, the study provides a unique information on the spectrum of treatment of all AF patients. ECG data are available from 2010 to 2018 of 86,500 patients, totally 1.3 million ECGs.

The complete information on purchases of all the medication on prescription will be used for the characterization of the population, and with the time span of 15 years, also trends in the medication use will be evaluated. The total number of purchases in this 411,080-patient cohort is remarkable—about 106 million purchases, and there have been e.g. 11.2 million purchases of beta blocking agents and 5.9 million purchases of oral anticoagulants. The changes in the use of antiarrhythmic medication, medications with known interactions with OACs, and the use of other cardiovascular medications will be evaluated.

A wide scale opportunity to analyze our cohort is enhanced with the data on income, education and socio-economic status and place of residence. It is well known that these aspects may have a marked impact on patients’ wellbeing, and for the first time this can be studied in Finnish AF population [7]. In principle, all the Finnish residents should receive all the treatments they need, paid by the taxation or at least taxation-based support (Beveridge model). However, it is well known that even in taxation-based health care arrangements patients with different income and socio-economic status are not treated equally, and income and socio-economic status are strongly associated with morbidity and mortality [18, 19]. With the information on places of domicile, regional differences of morbidity, mortality and use of resources can be elucidated.

Important substudies evaluates cost effectiveness of the given treatments. Data on contacts with the public Finnish health and social care systems will be utilized and completed with the data of private care reimbursement. The number of AF patients is increasing, and patients are getting older with higher number of co-morbidities. Therefore, the complete data and analyses of the treatments and their costs is crucial when the care pathways for the future are established.

### Strengths and limitations

The major strength of our study is its comprehensive nationwide data collection. AF patients are gathered from all the available nationwide registers with the background population of all Finnish residents [20]. To our knowledge, this is the only European nationwide AF study including also primary care patients [5]. Previous literature provides data on more-or-less selective AF populations, describing either data based on hospital registers, or regional health care delivery or insurance organizations, or are focused on certain age groups. In the present study, the cohort comprises patients from rural and urban areas, patients treated in hospital but also only in primary as well as private care regardless of the co-morbidities, age, income, or domicile. AF ablation is only provided by public health care in hospitals in Finland, and therefore we get also comprehensive data on this patient group.

The present study is completely based on the register data and is reliant on the general limitations of such approaches. Therefore, e.g. smoking habit, use of alcohol or height or weight are not available. Also, the data is as accurate as it is recorded. Fortunately, in particular Finnish special care register (HILMO) has a long history of high quality and is well validated and e.g. the diagnoses of stroke are reliably registered [8, 9]. Regarding the medication data, we are lacking information on purchase of acetylsalicylic acid while it is frequently bought over the counter without a prescription in Finland.

Does our register cover all Finnish AF patients? As noted, we are dependent on the coverage and the precision of the used registers. Therefore, we have only those patients, who have a diagnosis of AF documented in a register. Screening protocols increase the number of new AF patients, and the proportion of this increase have been as high as 60% [21]. However, in a thoroughly completed meta-analysis the number of new AF cases with a cross sectional screening seems to be quite limited and the mean percentage of new AF in screened cohorts older than 65 years was 1.4% [22]. In the light of our figures, that would yield approximately 10% more previously undiagnosed AF patients. Another minor possibility is that an AF patient has been treated without any remark of I48 diagnosis in the national health care registers. This could only occur if the treatment is given completely at a private clinic without any contact with public health care and without reimbursement of drug therapy for AF. Nevertheless, the prevalence of AF now documented—4.1% per the whole population—is the highest number recorded in a nationwide study.

## Conclusions

To obtain a complete analysis of a population one needs the complete cohort and the complete data from that population. The present study has one of the most comprehensive real-world data considering unselected AF patients based on nationwide register data using all available public resources. The number of Finnish AF patients was for the first time established, with a prevalence of 4.1% among Finnish population*.* Our findings will have guidance at least for national regimens and guidelines when AF patients are treated. Also, knowledge of cost-effectiveness as well as patients’ socio-economical profiles will markedly help evidence-based management in the care of increasing numbers of AF patients.

## Supplementary Information

Below is the link to the electronic supplementary material.

Supplementary file1 (DOCX 67 KB)

## Data Availability

Based on the contracts with the Finnish registries, the data are not available for sharing.
