# Supplementary material for: The nationwide Finnish anticoagulation in atrial fibrillation (FinACAF): study rationale, design, and patient characteristics
Source: Eur J Epidemiol. 2022 Jan 5;37(1):95–102. doi: 10.1007/s10654-021-00812-x (PMC8791884; doi:10.1007/s10654-021-00812-x)
Supplement: Supplementary file 1 — Supplementary file1 (DOCX 67 KB) [file 10654_2021_812_MOESM1_ESM.docx]

**The nationwide Finnish AntiCoagulation in Atrial Fibrillation (FinACAF): Study Rationale and Patient Characteristics**

**Appendices**

Mika Lehto, MD, PhD, Lohja Hospital, Department of Internal Medicine, Lohja, Finland; Heart and Lung Center, Helsinki University Hospital and University of Helsinki, Helsinki, Finland. ORCID ID 0000-0002-8691-5142

Olli Halminen, MSc, Aalto University, Espoo, Finland. ORCID ID 0000-0001-9266-843

Pirjo Mustonen, MD, PhD, Turku University Hospital and University of Turku, Finland. ORCID ID 0000-0003-1319-0248

Jukka Putaala, MD, PhD, Department of Neurology, Helsinki University Hospital and University of Helsinki, Helsinki, Finland. ORCID ID 0000-0002-6630-6104

Miika Linna, PhD, Professor, University of Eastern Finland, Kuopio, Finland; Aalto University, Espoo, Finland. ORCID ID 0000-0002-7660-2484

Janne Kinnunen, MD, Department of Neurology, Helsinki University Hospital and University of Helsinki, Helsinki, Finland.

Elis Kouki, BM, Heart and Lung Center, Helsinki University Hospital and University of Helsinki, Helsinki, Finland

Jussi Niiranen, MD, Heart and Lung Center, Helsinki University Hospital and University of Helsinki, Helsinki, Finland; Florida State University, Tallahassee, Florida, USA. ORCID ID 0000-0001-7751-6257

Juha Hartikainen, MD, PhD, Professor, Kuopio University Hospital and University of Eastern Finland. ORCID ID 0000-0003-0847-107X

Jari Haukka, PhD, Senior Lecturer, University of Helsinki. Finland. ORCID ID 0000-0003-1450-6208

K.E. Juhani Airaksinen, MD, PhD, Professor, Turku University Hospital and University of Turku, Finland. ORCID ID 0000-0002-0193-568X

Corresponding author: Mika Lehto, mika.lehto@hus.fi

Supplementary data: The Appendix provides: Definitions of the comorbidities, Primary Outcomes of the study, Characteristics of the entire study cohort, Characteristics of the entire study cohort in selected subgroups (age < 65 years, 65-74 years and ≥ 75 years, as well as females / males), Objectives of the study, and The list of names of the entire FinACAF study group.

**Appendix**

**Table 1e Definitions of the comorbidities**

|  | **ICD-10** | **ICPC-2** | **Laboratory^1^** | **Reimbursement code of KELA** | **ATC-code** |
| --- | --- | --- | --- | --- | --- |
| **Hyperlipidemia** | E78 | T93 | Chol > 5 mmol/l  Chol-LDL > 3 mmol/l | 206 | C10 |
| **Hypertension** | I10-I15 | K85  K86  K87 | - | 205 | C03A, C03B, C03DB, C03EA, C07A, C08CA, C08D, C09 |
| **Diabetes** | E10-E14 | T89  T90 | B -HbA1c ≥ 48 mmol/l | 103  215 | A10 |
| **Stroke** | I63-I64  I69.3-I69.8 | K90 | - | - | - |
| **Heart failure** | I50  I11.0  I13.0  I13.2 | K77 | - | 201 | \| - \| \| --- \| |
| **Vascular disease** | I20-I25  I65-I66  I67.2  I70 | K74  K75  K76  K91  K92 | - | 206 | - |

^1^ Used in those areas where laboratory information available

Diagnoses of the comorbidities are defined hierarchically:

- can be found as a diagnosis from one of the registers

- diagnostic code of entitlement to reimbursement

- medication: if the above sources do not include the diagnosis of hypertension, then the medical information is utilized for hypertension:

- C03A, C03B, C03DB, C03EA and C08CA (can also be used after the diagnosis AF)

- beta-blockers (C07) and diltiazem or verapamil (C08D) algorithm for diagnosis of hypertension:

- can be used at baseline only, i.e. before the diagnosis of AF. Cannot be utilized after the diagnosis of AF, because these are used in the treatment of AF.

- if a patient has a diagnose of coronary artery disease (I20-I25) then beta-blocker, diltiazem or verapamil cannot be utilized for diagnosing hypertension

- drugs acting on the RAAS system (C09) for the diagnosis of hypertension:

- can also be used after the diagnosis of AF

- if a patient has the diagnose of heart failure (I50) and / or cardiomyopathy (I420-I422, I429) then C09 cannot be utilized for diagnosing hypertension

- on the contrary, C09 cannot provide a diagnosis of heart failure, i.e. utilization of this drug group is limited to diagnosis of hypertension.

- laboratory results: hyperlipidemia and diabetes

**Table 2e Primary Outcomes**

| **Variable** | **Definition (ICD-10)** |
| --- | --- |
| Stroke/Transient ischemic attack | I63, I64, I693-I698, G45 |
| Myocardial infarction | I21, I22 |
| Other systemic thromboembolic events excluding stroke | I74, K550, N280 |
| Bleeding events | D500, D62, D683, I60-I62, I690-I692, I850, I864, J942, K221, K223, K226, K250, K252, K254, K256, K260, K262, K264, K266, K270, K272, K274, K276, K280, K282, K284, K286, K290, K625, K631, K633, K920-K922, N02, R04, R31, R58, S062-S066, S068 |
| Mortality: all-cause |  |
| Mortality: stroke, myocardial infarction, other embolic events, bleeding events | ICD codes as above |

**Table 3e** Characteristics at the time of entry to the study cohort of the atrial fibrillation patients in Finland 2004 - 2018, totally 411 080 patients.

|  | n (%) |
| --- | --- |
| Female | 201 042 (49%) |
| Age, mean (± SD), median; years | 73.2 (± 12.8), 75 |
| Age over 65 | 314 177 (76%) |
| Age over 75 | 205 244 (50%) |
| Hypertension | 314 152 (76%) |
| Diabetes | 84 990 (21%) |
| Stroke or TIA | 57 640 (14%) |
| Heart Failure | 75 346 (18%) |
| Vascular Disease* | 104 806 (25%) |
| Hyperlipidemia | 178 855 (44%) |
| CHADS_2_-VASc_2_, mean (± SD), median | 3.5 (±1.8), 4 |

* Coronary artery disease or peripheral artery disease.

**Table 4e** Characteristics at the time of entry to the study cohort of the atrial fibrillation patients in Finland 2004 - 2018, totally 411 080 patients. Patient groups: age < 65 years, 65-74 years and ≥ 75 years, as well as females / males.

|  | < 65 years  96 903 | 65-74 years  108 933 | ≥ 75 years  205244 | Female  201 042 (49%) | Male  210 038 (51%) |
| --- | --- | --- | --- | --- | --- |
|  | n (%) | n (%) | n (%) | n (%) | n (%) |
| Female | 27 257 (28%) | 46 422 (43%) | 127 363 (62%) | 201 042 (100%) |  |
| Age, mean (± SD), median; years | 55.2 (± 9.0), 58 | 70.3 (± 2.9), 70 | 83.2 (± 5.3), 83 | 77.1 (± 11.3), 79 | 69.5 (± 13.0), 71 |
| Hypertension | 63 096 (65%) | 86 046 (79%) | 165 010 (80%) | 162 994 (81%) | 151 158 (72%) |
| Diabetes | 14 251 (15%) | 25 083 (23%) | 45 656 (22%) | 40 237 (20%) | 44 753 (21%) |
| Stroke or TIA | 6 149 (6.3%) | 13 945 (13%) | 37 546 (18%) | 30 672 (15%) | 26 968 (13%) |
| Heart Failure | 8 464 (8.7%) | 14 458 (13%) | 52 424 (26%) | 40 950 (20%) | 34 396 (16%) |
| Vascular Disease* | 10 914 (11%) | 25 054 (23%) | 68 838 (34%) | 49 158 (24%) | 55 648 (26%) |
| Hyperlipidemia | 29 631 (31%) | 54 512 (50%) | 94 712 (46%) | 87 737 (44%) | 91 118 (43%) |
| CHADS_2_-VASc_2_, mean (± SD), median | 1.5 (±1.1), 1 | 3.1 (±1.3), 3 | 4.6 (±1.3), 4 | 4.3 (±1.5), 4 | 2.7 (±1.7), 3 |

* Coronary artery disease or peripheral artery disease.

**Objectives of the study**

Primary Objectives to investigate the:

1. Risk of stroke, systemic thromboembolism, bleeding events and myocardial infarction among AF patients in relation to different OAC treatments including warfarin treatment with the data of different TTR levels compared also with patients without any OAC treatment.

2. Risk of all-cause and cardiovascular death in relation to different OAC treatments including warfarin treatment with the data of different TTR levels compared also with patients without any OAC treatment.

Secondary Objectives to investigate the:

3. Use of health care services in relation to different OAC treatments.

4. Cost effectiveness in relation to different OAC treatments.

5. Relations of socio-economic status with treatments the patients are given, the quality of OAC as well as with the major outcomes.

6. Relations of presence of dementia and psychiatric illness with treatments the patients are given, the quality of OAC, as well as with the major outcomes.

7. Use of private health care system, cost and imbursement of it, and cost of reimbursed transportation services.

8. Risk of dementia in relation to different OAC treatments as well as with different socio-economic positions

9. Risk of stroke, systemic thromboembolism, myocardial infarction, and major bleeding events among AF patients after initiation of OAC treatment.

10. Risk of stroke, systemic thromboembolism, and major bleeding events in relation to time from AF diagnosis to time of initiation of OAC treatment.

11. Risk of stroke, systemic thromboembolism, myocardial infarction, and major bleeding events among AF patients who have stopped using OAC for any reason.

Exploratory Objectives to investigate the:

12. Incidence of anemia and renal impairment among AF patients in relation to different OAC treatments including warfarin treatment with different TTR levels.

13. Risk of stroke, systemic thromboembolism, myocardial infarction, and bleeding events among AF patients in relation to discontinuation of OAC treatment due to surgical operations and interventions.

14. How long it takes to achieve INR target in treatment initiators.

15. The quality of warfarin treatment (TTR level).

16. The use of antiarrhythmic medication in AF patients

17. The use of medications with known interactions with OAC, and the association of the use of these medications with the main outcomes.

18. The use of other cardiovascular medications in AF patients (ACE-inhibitors, ARBs, beta blocking agents, antiarrhythmics, cardiac glycosides, diuretics etc.), and the association of the use of these medications with the main outcomes.

19. The use of other medications with a meaningful role in AF patients (diabetes and lipid lowering medication, drugs for gastrointestinal acid related disorders etc.), and the association of the use of these medications with the main outcomes.

20. How long it takes to achieve elective cardioversion performed (procedure code TFP20) in OAC treatment initiators.

21. Risk of stroke, systemic thromboembolism, and bleeding events associated with different procedures (cardioversion, AF-ablation etc.).

22. The use of blood products in the study cohort.

23. risk of stroke, systemic thromboembolism, and bleeding events particularly in the subgroup of patients with diagnosis of cancer.

24. Recurrent stroke and bleeding risk. This will be done particularly in patients with co-morbidity of vascular disease (coronary heart disease, peripheral arterial disease, carotid/intracranial atherosclerotic disease).

25. ECG-database collected from the laboratory databases:

AF pattern:

- Only atrial fibrillation/flutter present in the ECGs

- Both AF and sinus rhythm present

From the last ECG with sinus rhythm:

- P-wave analysis (PA, PD, PPA V1, P-axis), PR-interval, (RAE, LAE, BAE);

From the last ECG:

- QRS duration, QTc, left ventricular hypertrophy, bundle branch blocks, late QRS transition, (R<S in V4), QRS axis, T-wave axis, T-wave amplitude (in V2-V6, I, aVL, aVF)

Prevalence of ECG findings in AF population and their correlations with endpoints are studied.

**FinACAF Study group (at present)**

Mika Lehto, MD, PhD, Lohja Hospital, Department of Internal Medicine, Lohja, Finland; Helsinki University Hospital and University of Helsinki, Helsinki, Finland

Juhani Airaksinen, MD, PhD, Professor, Turku University Hospital, University of Turku, Turku, Finland

Olli Halminen, MSc, Aalto University, Espoo, Finland

Pirjo Mustonen, MD, PhD, Turku University Hospital and University of Turku, Turku, Finland.

Jukka Putaala, MD, PhD, Department of Neurology, Helsinki University Hospital and University of Helsinki, Helsinki, Finland

Jari Haukka, PhD, Professor, Tampere University; Senior Lecturer, University of Helsinki

Miika Linna, PhD, Professor, University of Eastern Finland, Kuopio, Finland; Aalto University, Espoo, Finland

Aapo Aro, MD, PhD, Helsinki University Hospital and University of Helsinki, Helsinki, Finland

Fausto Biancari, MD, PhD, Helsinki University Hospital and University of Helsinki, Helsinki, Finland; Research Unit of Surgery, Anesthesia and Critical Care, University of Oulu, Oulu, Finland

Tuukka Helin, MD, PhD, Helsinki University Hospital and University of Helsinki, Helsinki, Finland

Jaakko Inkovaara, MD, Tampere University Hospital, Tampere, Finland

Saga Itäinen-Strömberg, MD, Helsinki University Hospital and University of Helsinki, Helsinki, Finland

Jussi Jaakkola, MD, PhD, Turku University Hospital, University of Turku, Turku, Finland

Santeri Jolkkonen, MD, University of Eastern Finland, Jyväskylä, Finland

Lotta Joutsi-Korhonen, MD, PhD, Helsinki University Hospital and University of Helsinki, Helsinki, Finland

Heini Jyrkilä, MD, Helsinki University Hospital and University of Helsinki, Helsinki, Finland

Kati Kaartinen, MD, PhD, Helsinki University Hospital and University of Helsinki, Helsinki, Finland

Ksenia Kalatsova, MD, Helsinki University Hospital and University of Helsinki, Helsinki, Finland

Elin Karlsson, BM, Helsinki University Hospital and University of Helsinki, Helsinki, Finland

Janne Kinnunen, MD, Helsinki University Hospital and University of Helsinki, Helsinki, Finland

Tuomas Kiviniemi, , MD, PhD, Turku University Hospital, University of Turku, Turku, Finland

Miikka Korja, MD, PhD, Helsinki University Hospital and University of Helsinki, Helsinki, Finland

Elis Kouki, BM, Helsinki University Hospital and University of Helsinki, Helsinki, Finland

Jaana Kuoppala, MD, PhD, University of Helsinki, Helsinki, Finland

Ossi Lehtonen, MSc, Aalto University, Espoo, Finland

Alex Luojus, MD, Helsinki University Hospital and University of Helsinki, Helsinki, Finland

Mikko Manninen, MD, PhD, Hospital Orton Ltd, Helsinki, Finland.

Leena Martola, MD, PhD, Helsinki University Hospital and University of Helsinki, Helsinki, Finland

Keijo Mäkelä, MD, PhD, Turku University Hospital, University of Turku, Turku, Finland

Leena Ristolainen, PhD, Hospital Orton Ltd, Helsinki, Finland.

Jussi Niiranen, MD, Heart and Lung Center, Helsinki University Hospital and University of Helsinki, Helsinki, Finland; and Florida State University, Tallahassee, Florida, USA

Timo Nuutinen, MD, Helsinki University Hospital and University of Helsinki, Helsinki, Finland

Tero Penttilä, MD, PhD, Tampere University Hospital, Tampere, Finland

Jarno Satopää, MD, PhD, Helsinki University Hospital and University of Helsinki, Helsinki, Finland

Paula Tiili, MD, Helsinki University Hospital and University of Helsinki, Helsinki, Finland

Konsta Teppo, MD, Turku University Hospital, University of Turku, Turku, Finland

Anita Ylimäki, MD, Helsinki University Hospital and University of Helsinki, Helsinki, Finland

Mika Niemelä, MD, PhD, Professor, Helsinki University Hospital and University of Helsinki, Helsinki, Finland

Juha Hartikainen, MD, PhD, Professor, Kuopio University Hospital, University of Eastern Finland, Kuopio, Finland

Mikko Niemi, MD, PhD, Professor, University of Helsinki, Helsinki, Finland
